# Staff and Users’ Experiences of Pharmacy-Based Sexual and Reproductive Health Services: A Qualitative Interview Study from the UK

**DOI:** 10.3390/pharmacy8040206

**Published:** 2020-11-03

**Authors:** Julia Gauly, Jonathan Ross, Joanne Parsons, Helen Atherton

**Affiliations:** 1 Warwick Medical School, University of Warwick, Coventry CV4 7HL, UK; Jo.Parsons@warwick.ac.uk (J.P.); H.Atherton@warwick.ac.uk (H.A.); 2 University Hospitals Birmingham NHS Foundation Trust, Birmingham B15 2TH, UK; Jonathan.Ross@uhb.nhs.uk

**Keywords:** contraception, sexually transmitted infection(s), sexual and reproductive health, pharmacy staff, pharmacy users, qualitative study, public health, service delivery, United Kingdom, thematic analysis

## Abstract

Since August 2015, a large range of sexual health and reproductive health services have been provided in more than 120 pharmacies across Birmingham (England). Our study aimed to explore how pharmacy staff and pharmacy users experience delivering or being provided with sexual health and reproductive health services. Between March and September 2019, semi-structured interviews were conducted with 15 pharmacy staff delivering sexual and reproductive health services and 15 people who had used a sexual and reproductive health service at the pharmacy. Interviews were analysed thematically. Pharmacy users found services convenient to use and were largely satisfied with pharmacy staff consultation skills. Staff were motivated to deliver the services, although some felt that they did not receive sufficient recognition for their work. Barriers to pharmacy-based sexual and reproductive health services were identified, including lack of privacy for users, lack of staff and user awareness of the services, lack of trained staff to deliver services and lack of capacity for copper coil insertions in females presenting for emergency contraception. The identification of barriers to effective service provision can be used to improve the delivery of sexual and reproductive health services in pharmacies and lead to a greater uptake.

## 1. Introduction

It is estimated that one million new urogenital sexually transmitted infections (STIs) are contracted each day, affecting the health and lives of people worldwide [1,2]. Furthermore, high rates of unintended pregnancies leading to abortion remain a global public health challenge [3]. Sexual and reproductive health are also major public health concerns in England, where STI diagnoses in 2018 rose by 5% compared to the previous year, and the percentage of conceptions leading to abortion amongst females in England and Wales increased from 22.7% to 24% in the same time period [4,5,6].

Following the Health and Social Care Act in 2013, local authorities in England have been responsible for the planning, purchasing and monitoring of most sexual and reproductive health services, including contraception, and prevention and testing for HIV and other STIs [7,8]. While local authorities are legally required to provide those services, they are free to decide which providers should deliver what services [9]. With more than 11,500 pharmacies across England, local authorities have increasingly recognised their potential to increase access to sexual and reproductive health services [10].

A recent survey shows that most local authorities provide emergency contraception, chlamydia screening and condoms through pharmacies but that only a few deliver additional sexual and reproductive health services [11]. The local authorities in Birmingham, England’s second largest city and, as of 2018, a city with higher STI rates than the national average, however, have been more ambitious and provide a large range of pharmacy-based sexual and reproductive health services through *Umbrella*.

### 1.1. Umbrella and Their Pharmacy Services

In 2014, the University Hospitals Birmingham (UHB) NHS Foundation Trust was awarded a tender by Birmingham City Council and Solihull Metropolitan Borough Council (local authorities in the West Midlands) to deliver an innovative and integrated sexual and reproductive health service (SRHS) in Birmingham. In the following year, the SRHS branded *Umbrella* was launched [12]. *Umbrella* offers a range of prescription-free and cost-free services (as they are deemed NHS services) through several pathways, including pharmacies. As of 2020, more than 169 pharmacies are delivering *Umbrella*’ services.

Public Health England, an executive agency of the Department of Health and Social Care in the UK, encourages health providers to publicise access to sexual and reproductive health services. In line with this guidance, *Umbrella*’s communication team promotes its pharmacy-based sexual and reproductive health services amongst others on social media, on the *Umbrella* website and posters across the city. *Umbrella* pharmacies also have posters advertising the *Umbrella* services hanging in their stores and in pharmacy store windows.

Pharmacy based *Umbrella* services have operated at what are known as Tier 1 or Tier 2 levels. Both Tier 1 and Tier 2 pharmacies offer emergency contraception pills, and schedule coil fitting appointments for females presenting for emergency contraception at the closest sexual health clinic. Tier 2 pharmacies can additionally provide advance provision of the emergency contraceptive pill for future use. Both Tier 1 and Tier 2 pharmacies also dispense condoms and provide self-sampling kits testing for up to five different STIs (chlamydia, gonorrhoea, syphilis, HIV and hepatitis B) that can be pre-ordered on *Umbrella*’s website. Self-sampling kits testing for chlamydia and gonorrhoea are also offered to females presenting for emergency contraception. In contrast to Tier 1 pharmacies, Tier 2 pharmacies have STI self-sampling kits in stock and can supply them immediately to pharmacy users without pre-ordering. A vulvovaginal swab (for females) or a urine collection pot for males is used to test for chlamydia and gonorrhoea. Rectal and oral swabs are included depending on sites of exposure. To test for syphilis, HIV and hepatitis B, lancets and blood-sample collection tube is provided. Pharmacy users who receive an STI self-sampling kit are provided with written instructions and access to online video instructions.

Tier 2 pharmacies also provide chlamydia treatment, and can initiate the contraceptive injection or oral contraception. An overview of all *Umbrella* pharmacy services and eligibility criteria is provided in Table 1.

On behalf of *Umbrella*, all service activity and the demographic information of service users are recorded by pharmacists on a web-based system called PharmOutcomes^®^ (Pinnacle Health Partnership LLP, East Cowes, England). Information on service users who pre-order STI self-sampling kits is collected through an online patient questionnaire located on *Umbrella*’s website.

*Umbrella* pharmacies are required to have a private consultation room and services can only be delivered by pharmacists who have undergone *Umbrella*’s training. Training for the Tier 2 level is more comprehensive than Tier 1 level training. Condoms and STI self-sampling kits can also be delivered by pharmacy healthcare assistants after they have received appropriate training from *Umbrella*. Healthcare assistants collect data for these services on paper forms, and the data are later entered into PharmOutcomes^®^ by pharmacists.

### 1.2. Aim of This Study

To date there is limited research on staff and users’ experience of delivering or being provided with pharmacy-based sexual and reproductive health services [13]. Furthermore, no previous qualitative study has looked at a pharmacy service delivering a wide range of sexual and reproductive health services [13]. Only a limited number of studies have included pharmacy healthcare assistants’ views on the delivery of pharmacy-based sexual and reproductive health services, although they are often the first point of contact for pharmacy users [14,15,16,17].

The aim of this study was therefore to explore pharmacists’ and pharmacy healthcare assistants’ experiences of delivering sexual health and contraception services, and to explore pharmacy users’ experiences of using these services.

## 2. Materials and Methods

Ethical approval was received from the South Central—Oxford B Research Ethics Committee and from the Health Research Authority prior to data collection (REC Reference: 18/SC/0511). Additionally, approval from the University Hospital Birmingham NHS Foundation Trust was obtained prior to the beginning of the study. Informed oral consent was obtained from all participants prior to interviews commencing. This study is reported following the Standards for Reporting Qualitative Research [18].

### 2.1. Study Participants

Pharmacists and pharmacy healthcare assistants were eligible to take part in the study if they were trained to deliver *Umbrella*’s services and working at a pharmacy delivering *Umbrella* services. Pharmacy users were eligible to take part if they were at least 16 years old and had accessed at least one of the *Umbrella* services at a pharmacy. Ideally, participants would have been sampled according to a large range of parameters (e.g., ethnicity, religion and age). However, sexual and reproductive health is a sensitive topic and it was considered that people may not have been willing to provide personal information prior to being interviewed. It was therefore decided to keep the information requested prior to the interview to a minimum and only sample according to a number of factors that would allow to ensure some variety in candidates [19]. Relevant parameters for staff included the following: (1) service level offered at *Umbrella* pharmacy employed at (Tier 1 and/or Tier 2), (2) *Umbrella* pharmacy store employed at (e.g., independent/chain pharmacy and location of pharmacy) and (3) gender of pharmacy staff. Relevant parameters for pharmacy users included the following: (1) type of sexual and reproductive health service requested at *Umbrella* pharmacy (e.g., emergency contraception and chlamydia treatment), (2) *Umbrella* pharmacy store (e.g., independent/chain pharmacy and location of pharmacy) and (3) gender of pharmacy user).

### 2.2. Recruitment

Letters inviting *Umbrella* pharmacies to volunteer to support the recruitment of the study by distributing flyers to their staff and pharmacy users were sent via email to all *Umbrella* pharmacies by the chair of the local pharmaceutical committee.

Eight *Umbrella* pharmacies agreed to support study recruitment. With these eight pharmacies via a Schedule of Activities, a document which can be used with participating organisation as a form of site agreement which is provided by the Health Research Authority. With regards to the recruitment of pharmacy staff, pharmacies were free to decide whether to directly hand out flyers to pharmacy staff or leave the flyers in a common room for pharmacy staff. With regards to pharmacy users, staff were asked to hand out flyers to those who had been provided with an *Umbrella* service at their pharmacy. Some of the eight pharmacies who supported the recruitment belonged to different pharmacy chains (*n* = 4), whereas the others (*n* = 4) were independent pharmacy stores. All of the pharmacies had a different postcode, and while some of the pharmacies were located in the city centre (*n* = 3), others were located outside the city centre (*n* = 5). Locations of the pharmacies included shopping malls, shopping centres, the high street and freestanding buildings.

Those staff members and pharmacy users who had received a flyer and were interested in taking part in the study could then contact the first author, using the contact details provided on the flyer. Interested candidates were asked for some details to allow to sample participants purposively (see Section 2.1) and provided the study information. Telephone interviews were scheduled with eligible candidates who met the sampling frame and agreed to take part.

Additionally, pharmacy staff were recruited from training events held by *Umbrella*’s Education Team in Birmingham. Interested candidates were asked to provide their contact details. Interested candidates were contacted by the first author the following day, asked for some details to allow to sample participants purposively (see Section 2.1) and provided with the study information. With those who were still interested in taking part and met the sampling frame, interviews were scheduled.

Pharmacy users were also recruited via social media posts, using Twitter and Facebook to raise awareness about the study. Interested candidates could then contact the first author using the contact details provided in the posts and Tweets. *Umbrella’s* communications team also set up a secure web page where candidates could register their interest in taking part. The link to the pre-registration site was located in several areas within *Umbrella’s* website and on the website of the University Hospital Birmingham NHS Foundation Trust. The link was also shared on Twitter and Facebook by the first author and *Umbrella*’s communications team. Candidates who had pre-registered their interest in taking part were then contacted by the first author, asked to provide some information to allow for the purposive sampling of candidates (see Section 2.1) and provided with the study information. Interviews were scheduled with pharmacy users who met the eligibility criteria and sampling frame and agreed to participate after having considered the study information.

The participation in the interview study was voluntary and had no impact on staff and users’ access to delivering or being delivered with *Umbrella*’s services. Participation in this study was confidential and the health provider *Umbrella* was not informed about which pharmacies and pharmacy staff members were participating in the research.

**Figure 1:**
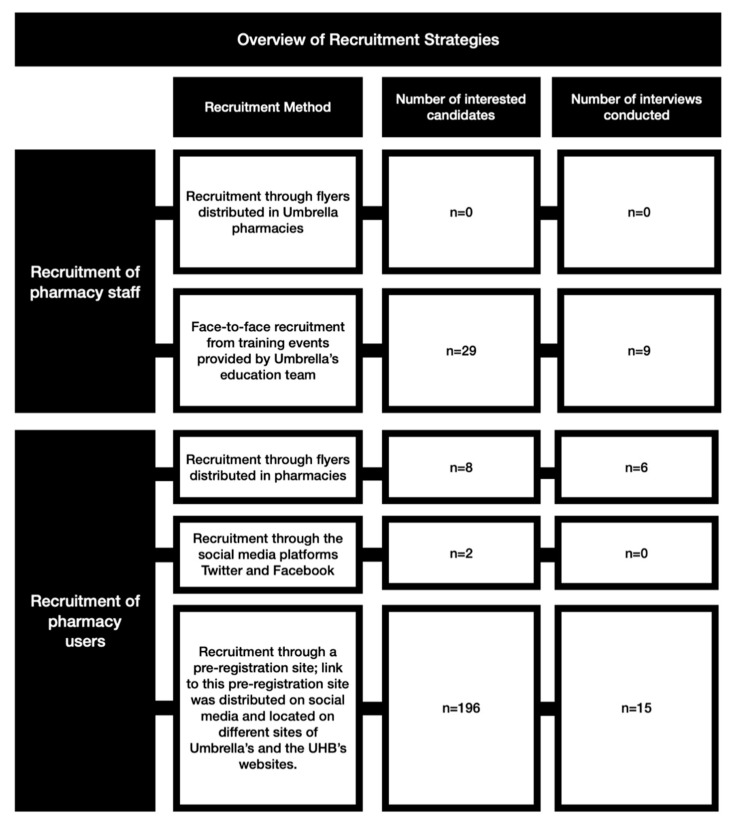
Overview of recruitment strategies.

An overview of the recruitment is provided in Figure 1.

### 2.3. Data Collection

All interviews were conducted by the first author, a female researcher trained in qualitative interviewing, over the phone. Example questions from the topic guide, which was informed by our recent systematic review [13], are provided in Table A1. Demographic characteristics that have previously been shown to impact peoples’ attitudes towards the sexual and reproductive health services were collected at the end of the interview in order to give context to the data. These characteristics included age [20], gender [20], ethnicity [21] and religion [21,22]. All study participants were provided with a £10 shopping voucher as an appreciation for their time. In line with the ethical approval, potential study participants were informed that they would receive a £10 voucher as thank you for their participation on advertising materials (e.g., social media posts and flyers). The voucher was emailed to study participants after the interview had been conducted.

All interviews were audio-recorded on encrypted devices and anonymised prior to the transcription, which was conducted by a professional transcription service. Data collection was discontinued once data saturation was reached, defined as the point where no new themes emerged from the data [23,24].

### 2.4. Data Analysis

All transcripts were checked for accuracy by the first and the last author. Next, all interview transcripts were uploaded into the software programme NVivo v12. Modified grounded theory was used as theoretical framework of the study [25]. This involved coding the data using an inductive approach, meaning that codes were derived from the data. In addition, it was cross checked whether codes came from the systematic review which informed this study [13]. It was also analysed whether differences in experiences appeared to be related to participants’ demographic characteristics by comparing participants’ data according to their age, gender, ethnicity and religion. The research analysis was a reflexive and iterative process, which involved the development of a coding framework. To reduce bias of the thematic analysis, the third author independently coded 30% of all interviews using the same approach as the first author and developed a separate coding framework. To increase the validity of the thematic analysis, all authors discussed the two coding frameworks to produce one final coding framework. Themes were then identified, reviewed and defined iteratively. Finally, the themes and subthemes were presented in form of text and mind maps. Quotes were used to substantiate analytic findings.

## 3. Results

Overall, 15 pharmacy staff (nine pharmacists and six pharmacy healthcare assistants) and 15 pharmacy users were interviewed in total. In-depth interviews revealed that, for our participants, gender, ethnicity, age or religion did not affect their experiences. Demographic details are presented in Table 2 for pharmacy staff and Table 3 for pharmacy users.

**Figure A1:**
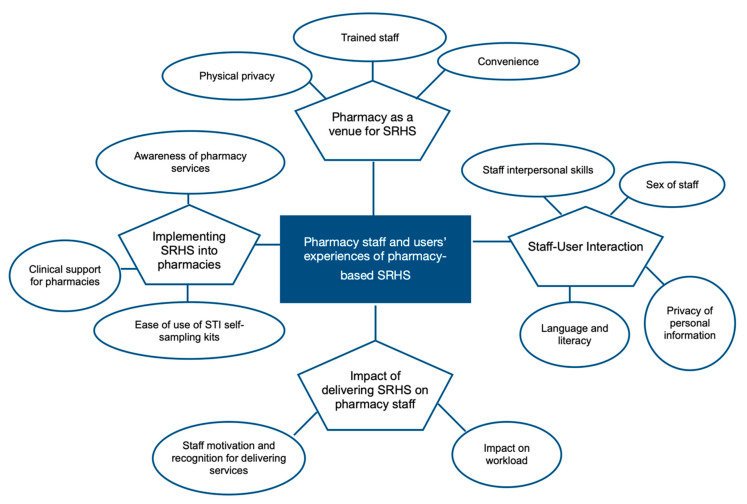
Overview of themes and subthemes.

Four main themes relating to pharmacy staff and pharmacy users’ experiences were identified: staff-user interaction; pharmacy as a venue for sexual and reproductive health services (SRHS); impact of delivering SRHS on pharmacy staff; and implementing SRHS in pharmacies (Appendix A Figure A1). These will each be described in turn, illustrated with examples of direct quotes from the transcripts.

### 3.1. Theme 1: Pharmacy as a Venue for SRHS

This theme is focused on factors related to the uniqueness of the pharmacy as a venue for SRHS. The following sub-themes were identified: physical privacy, convenience and trained staff.

#### 3.1.1. Physical Privacy

The consultation room provided most pharmacy users with sufficient privacy during the service delivery, the only disconfirming case being one pharmacy user who criticised that the consultation room was not soundproof. However, a lack of physical privacy was a greater issue for people when initially requesting an SRHS. Physical privacy was perceived as inadequate where staff were not discreet, the pharmacy was busy, the counter was not in a separate area and parallel queues were used within the pharmacy. Several pharmacy users expressed that they felt embarrassed attending for an SRHS and were concerned about being judged by other pharmacy clients or pharmacy staff:

“*There is definitely a feeling of judgement when you’ve got people that are stood behind you in the queue and whatnot and you’re asking for the morning after pill*”

(pharmacy user, female, age group: 25–29).

Some pharmacy users were expressing that they were accessing a separate pharmacy for sexual and reproductive health concerns as they feared being judged from their family pharmacy staff:

“*So that’s why I didn’t choose to go in there, because it’s more of a judgement element to be honest, because, because I’m waiting for so long for the coil to, you know, get that appointment ... readily available ... I had to go into that pharmacy three weeks for the same thing (emergency contraception). And that’s not because I’m not, being careless, I’m using things, they’re just not working*”

(pharmacy user, female, age group: 25–29, ID number: 1082).

Hence, a high need for privacy which was in some cases linked to self-perceived stigma associated with requesting SRHS was found to be important to users. Some pharmacy users avoided using *Umbrella*’s services at pharmacies either where they knew staff, where they suspected they would be seen by other people that they knew or where other pharmacy clients were present.

“*Sometimes when I go into my other pharmacy I do have to wait until people have gone out, ‘cause you don’t necessarily wanna be discussing that in front of other people, do you know what I mean? It’s quite sensitive*”

(pharmacy user, female, age group 25–29).

#### 3.1.2. Convenience

One advantage of pharmacy based sexual health services was that they were accessible, for example that they were easy to reach from work or home:

“*’Cause I get, I live down the road to that pharmacy ... so it’s very convenient of me to go up there*”

(pharmacy user, female, age group: 25–29).

Further, pharmacy users appreciated that pharmacies had long opening hours, short waiting times and that no appointments needed to be booked in advance:

“*I went back to the same pharmacy and saw a female pharmacist, because both times it was just, just really straight forward, you didn’t need an appointment, was seen really, really quickly, and the staff were nice, and it was just way more, I suppose convenient*”

(pharmacy users, female, age group: 18–24).

#### 3.1.3. Trained Staff

Not always having pharmacy staff trained to deliver *Umbrella*’s services was a barrier to SRHS. Pharmacy healthcare assistants described that some pharmacy users got upset when not being able to access time sensitive services such as emergency contraception:

“*I was like, ‘I’m really sorry, but we haven’t got a pharmacist who can do that service for you’. And she got quite upset. You know, you know, she, she was, like, quite teary. And I’m like, you know, ‘If there was something I could do for you, I would’. But she was ... I think she was like, you know, she just felt she needed it there and then*”

(pharmacy healthcare assistant, female, age group: 30–39).

Pharmacy healthcare assistants described that they found it frustrating that they had to turn people away because a trained pharmacist was not available:

“*So it just, it’s frustrating we have to turn people away because we haven’t got the right pharmacist in*”

(pharmacy healthcare assistant, female, age group: 30–39).

### 3.2. Theme 2: Staff-User Interaction

This theme is focussed on issues relating to the interaction between pharmacy staff and pharmacy users. Four sub-themes were identified: sex of staff; staff interpersonal skills; privacy of personal information; and language and literacy.

#### 3.2.1. Sex of Staff

Whilst not true for all pharmacy users, many had a preference regarding the sex of staff delivering the SRHS. Females, particularly, preferred to be counselled by a female, and some stated that the lack of a female pharmacist would prevent them from approaching the pharmacy for sexual-health services.

“*I dunno, I don’t think I’d be comfortable. If there wasn’t a lady working there I probably wouldn’t go to that place*”

(pharmacy user, female, age group 25–29).

Some pharmacy users specifically asked at the counter for a female pharmacist. A male pharmacist stated that he lets his female pharmacy healthcare assistants convince females to speak to the male pharmacist. Further, a male pharmacy healthcare assistant reported that the pharmacist offered female pharmacy users to be chaperoned by female pharmacy healthcare assistants and that this was, in some cases, accepted by females who originally wanted to speak to a female pharmacist.

“*There’s a, a lot of Healthcare Assistants there. So they can chaperone with the pharmacist. If they’re happy to go with the pharmacist then they can be chaperoned*”

(pharmacy healthcare assistant, male, age group, 40–49).

While same sex delivery was found to be important, some users wanted to be counselled by the opposite sex. While some pharmacy users did not indicate a reason for this, one male participant stated that he would rather be counselled by a female, as he was attracted to males.

“*’Cause I’m not attracted to females, if that makes sense? So it’s like, a bit weird saying it to the sex I’m attracted to, if that makes sense?*”

(pharmacy user, male, age group, 18–24).

#### 3.2.2. Staff Interpersonal Skills

Pharmacy users, on the whole had a positive experience of interactions with pharmacy staff and often described them as friendly and sensitive. However, a there were some pharmacy users who had negative experiences with pharmacy staff whom they perceived to be not confident, not respectful of users’ choices or judgemental.

“*I had an infection and then I’d, I’d had intercourse again and then there was a, there was a slip-up with the condom and then I had to go back. And he’s, and then, at the end of him seeing me, he said, ‘I don’t want to see you back here again’. And that, that was a few years ago, but it always has stuck with me ... because that was really upsetting*”

(pharmacy user, female, age group: 18–24).

Pharmacy staff were aware that demonstrating confidence was important when delivering services. Some reported that they would have liked more training, and in particular more roleplaying experience in order to gain confidence.

“*I think we could have done with a little bit more training and probably a bit more roleplaying. And just to, yeah, I think they could have done with a little bit more training, just so that you are more confident in providing every service*”

(pharmacist, female, age group: <30).

#### 3.2.3. Privacy of Personal Information

The acceptability of having to provide personal information (e.g., name, date of birth and sexual history) varied widely amongst study participants. There was evident confusion about the need for collection of this data. Some pharmacy staff and pharmacy users felt that the information requested was relevant, while others were not sure why provision of personal information was needed. Further, some pharmacy users felt uncomfortable being asked for personal information due to uncertainty about what would happen with the information and due to privacy concerns.

“*I just felt uncomfortable to be honest ... that’s just me personally. I like to be a private person*”

(pharmacy user, female, age group: 25–29).

Dealing with pharmacy users’ discomfort was a challenge for pharmacy staff who also reported that some pharmacy users preferred to buy emergency contraception rather than use the free *Umbrella* service to avoid the need to provide personal data.

“*I’ve actually had patients who won’t go for the Umbrella service, just because of that, and they’d ... prefer to buy it I’ve actually had customers, not many, but there are a few customers that, even after we reassure them that all this information is confidential, just don’t like the idea of giving their names and date of births*”

(pharmacist, male, age group <30).

There were mixed views on whether personal health records should be shared between the pharmacy and *Umbrella* clinics. While some pharmacy staff members felt that this may be useful, they believed that pharmacy users would not want their data to be shared. Some users actually expected pharmacies to share the data with *Umbrella* clinics. Some users stated that if pharmacies already had the data, then that would provide them with more reassurance.

“*If it was like, the clinic could share the information with the pharmacy it felt like it wouldn’t be necessary, if that makes sense? You know, like, the database you keep all the information on? Like, if the pharmacy had access to that as well, it would just your name, date of birth and address, if that makes sense? It would be less, well, anxious…*”

(pharmacy user, male, age group: 18–24).

#### 3.2.4. Language and Literacy

Several pharmacists had difficulties delivering SRHS to people who were not fluent in English or illiterate:

“*There can sometimes be barriers, for example language barriers, if I cannot understand what somebody’s saying, I cannot actually provide a service…I have had a few incidents of that*”

(pharmacist, female, age group: <30).

Some pharmacists used *Google translate* and *Google images* to explain SRHS services. Pharmacists recognised the limitations of information sheets as they were of no use to illiterate pharmacy users. Where pharmacists were not able to communicate the services appropriately, they referred pharmacy users to the closest sexual health clinic:

“*Cause sometimes when they’re a bit, have a bit of broken English it’s a bit harder, and that’s when I maybe refer them to (name of sexual health clinic), or something like that. But I try and use Google Translate as much as I can*”

(pharmacist, female, age group 30–39).

### 3.3. Theme 3: Implementing SRHS into Pharmacies

This theme relates to the way that SRHS are implemented into the pharmacy. Three subthemes were identified: awareness of pharmacy services; clinical support for pharmacies; and ease of use of STI self-sampling kits.

#### 3.3.1. Awareness of Pharmacy Services

Staff and users who participated in a semi-structured interview felt that there was a lack of awareness of pharmacy-based SRHS and a lack of clarity about what they entailed. Interviews with both pharmacy staff and users showed a lack of awareness of the full range of *Umbrella’s* services and there was confusion amongst pharmacy users about where the services were offered, and who could access the services in terms of age and sex. Pharmacy staff felt that the eligibility criteria of services were not clearly advertised.

Further, a user reported that a pharmacy healthcare assistant had not been aware that *Umbrella*’s services are free of charge for all females between 13 and 60.

“*And so then I, I had to, to pay that charge and then I got the emergency contraception from her. They do, they’ve got the Umbrella service, they’ve even got the ... ‘cause I was, ‘cause I was quite shocked myself ... because it does say, online it does say that it’s free ... and they’ve even got the posters, they’ve even got the sticker and they’re part of Umbrella. And then she said there’s a 20-something pound charge*”

(pharmacy user, female, age group: 25–29).

Some users also stated that online information was not up to date making it difficult to find out where they could access the services.

“*Some of the pharmacies that are listed on the website, it’s quite dated, so I gave a few a call and they said they no longer supply that, the Umbrella services, but those websites are still ... those pharmacies are still on the website*”

(pharmacy user, female, age group 18–24).

As a result of this users had to call the pharmacy in advance to check whether the service and a trained pharmacist was available.

#### 3.3.2. Clinic Support for Pharmacies

*Umbrella* has a clinical support team, which can provide clinical advice to pharmacy staff and schedule clinical appointments for females presenting in in the pharmacy for emergency contraception. While staff appreciated having this advice line, they sometimes experienced difficulties getting through to the clinical support team.

“*And, especially on weekends, it can be really, really difficult to get through as well, and especially when you’re not sure what to do in this situation, you need some guidance and the customer’s waiting as well, it can be really, really frustrating*”

(pharmacist, male, age group: <30).

Further, pharmacy staff felt that there often were not sufficient clinical appointments available for females wishing to get a copper coil inserted for emergency contraception:

“*So then I’ve gotta go on the computer and try and get them an appointment, and it’s so difficult to ... find them an appointment, to the point where I’ll be weeks away. So that’s another difficulty I face as well, just trying to get them an appointment at one of the Umbrella clinics*”

(pharmacist, male, age group: <30).

#### 3.3.3. Ease of Use of STI Self-Sampling Kits

Many pharmacy users described difficulties conducting the blood test that is part of the STI-self sampling kit. Some were unable to complete the test and instead preferred to get tested at a clinic. Users felt that not sufficient lancets were provided in the testing kit and that the instructions were not clear enough.

“*There’s text in it…so with me I confuse which is which, because I don’t understand what I’m doing. And I don’t know which one I’m doing where, and what I’m doing which, though. If they specified that a little bit more better, then I might be able to continue using that service*”

(pharmacy users, transgender woman, age group: 25–29).

Pharmacy staff were aware that users had difficulties with the blood test. They found that they could support users with the blood sample in the pharmacy and users welcomed this idea. Pharmacy staff felt that the blood test could be supported by pharmacy staff.

“*So I think they don’t want to take that on themselves, they want someone else to do that for them. Which I think a pharmacist is ideally placed to do so*”

(pharmacist, female, age group: <30).

### 3.4. Theme 4: Impact of Delivering SRHS on Pharmacy Staff

This theme relates to the impact that the delivery of SRHS has on staff. Two subthemes were identified: Impact on workload and; staff motivation and recognition for delivering services.

#### 3.4.1. Impact on Workload

Given that pharmacists were responsible for directly providing most sexual and reproductive health services themselves, their workload was more affected than pharmacy healthcare assistants. The consultation and the collection of personal information from pharmacy users were perceived as time consuming and sometimes associated with increased stress and time pressure.

Pharmacists felt that it would be helpful if pharmacy users could pre-register themselves so that the collection of personal information could occur prior to the consultation which would be time saving and also ensure that the consultation time could be spent on the patients’ needs.

“*And so if it could speed up the process of having them pre-registered on the system then that would cut the consultation down in half. And then I could spend longer then actually, like I said, identifying maybe the patient’s unknown needs rather than just the immediate concern*”

(pharmacist, male, age group: 30–39).

Pharmacy healthcare assistants have to collect information on pharmacy users on paper forms and pharmacists then enter the information onto PharmOutcomes^®^. Pharmacy staff felt that this procedure duplicated work and felt that it would be easier if pharmacy healthcare assistants could also record data electronically. In agreement with their pharmacist, some pharmacy healthcare assistants were already entering data on PharmOutcomes^®^ although they were not supposed to.

“*When the pharmacist was in ... we would just say, ‘Oh’, you know, ‘we’ve done this service. Can we put it on PharmOutcomes?’ The pharmacist would be, ‘Yes’, you know. ‘Yeah, that’s fine. Just go in and put it on*’”

(pharmacy healthcare assistant, female, age group: 30–39).

Some pharmacists found it difficult to embed the delivery of SRHS within the rest of their duties due to time pressure. Another challenge for pharmacists was that they could not plan ahead when pharmacy users were coming for an SRHS. These pharmacists suggested introducing an appointment system, which would alleviate their difficulty in fitting additional work within their existing job role.

“*Well I think it would be great to, sort of, have a place where certain bookings were made, maybe, so it could be a more controlled process and it wasn’t just that people are coming up on the day and saying that ‘We need to have this service*’”

(pharmacist, female, age group: <30).

There was no difference between the perception of workload between Tier 1 and Tier 2 pharmacies. Instead, the perception of workload appeared to be associated with the busyness of the pharmacy and staffing levels. Pharmacists found it difficult to deliver services when staff levels were low or when they were alone in the pharmacy. Low staffing levels were also found to be a reason why some pharmacists could not provide the more comprehensive Tier 2 service. Some pharmacists expressed that they were unable to deliver any additional services, indicating that their workload was at the maximum:

“*But we’re not staffed to a great level. This is why ... somebody’s always asked me, ‘Why aren’t you a Level, Tier 2 pharmacy?’ and I tell them, ‘It’s because I, I couldn’t just, I, I, I can’t, I couldn’t do that service in my pharmacy. It’ll take too much, there’s too much time pressure and staffing pressures on, on my, on my staff that I wouldn’t be able to run a Tier 2 service’*”

(pharmacist, male, age group: 30–39).

#### 3.4.2. Staff Motivation and Recognition for Delivering Services

Pharmacy staff were generally motivated to deliver SRHS and many staff members stated that it increased their job satisfaction, including because being trained for SRHS made them more employable:

“*I guess for me, personally, in offering the Umbrella services it does mean that you’ll be more employable. So say if I went, so when I’m an actual pharmacist, if I’m locum-ing at, like, different pharmacies I guess the fact that you offer them services does, kind of, make you more employable to various pharmacies if you are trained in a number of services*”

(pharmacy healthcare assistant, female, age group: <30).

Staff were also grateful to be in a position to alleviate pressure on the healthcare system and to help people.

“*I think the best thing about delivering sexual health services is being able to be in the position to help somebody that is not happy about what’s happened or maybe gets in an accident and they, they’re quite worried, they’re quite anxious, they don’t know how to feel*”

(pharmacist, female, age group: <30).

While pharmacy staff were motivated to deliver services, they felt that they did not receive sufficient recognition for developing beyond their traditional role. While pharmacists were more like to state that pharmacy users were recognising their work, several pharmacy healthcare assistants felt that pharmacy users were distrusting towards them and did not recognise them as healthcare professionals:

“*Patients and customers don’t see the pharmacy team as professionals, as they would the pharmacist. So they’ll trust more what the pharmacist is saying than the pharmacy advisor*”

(pharmacy healthcare assistant, female, age group: 30–39).

However, pharmacy healthcare assistants described that wearing the *Umbrella* name badge made them more approachable. Pharmacy healthcare assistants also felt that they should receive financial recognition for their training. Both pharmacists and pharmacy healthcare assistants felt that they were not involved in decisions relating to the delivery of pharmacy-based SRHS and were not asked enough for feedback.

“*Because we’ve never been asked for feedback. I mean, we get mystery shopped. But we don’t really get asked, like, you know, what else could we possibly do to improve the services, or, you know, what do you think. We don’t really get asked that, to be honest*”

(pharmacy healthcare assistant, female, age group: 30–39).

## 4. Discussion

To our knowledge, this is the first study to explore pharmacists’, pharmacy healthcare assistants’ and pharmacy users’ experiences of a wide range of pharmacy-based SRHS.

Consistent with previous research, the findings of our study suggest that pharmacies are convenient to use [13,26,27]. Pharmacies should therefore consider maintaining features such as long opening times and walk-in services, which are perceived as convenient by pharmacy users. Those commissioning sexual and reproductive health services should consider how best to integrate and expand pharmacy provision within their service delivery model.

In accordance with studies on different pharmacy services [28], our study showed that lack of privacy was a concern for pharmacy users, especially when the pharmacy was busy. This suggests that such concerns are not unique to SRHS but to pharmacy services in general. Further, our study supports existing evidence that suggests that the pharmacy layout, e.g., the queuing system and the location of the pharmacy counter, can enhance or hinder the perceived level of privacy [29]. While the use of a consultation room helped addressed privacy during the consultation, one pharmacy user complained that the room was not soundproof. That privacy issues can remain even when using private consultation rooms was also found in a previous study [30]. According to NHS England, it should not be possible to be overheard in the pharmacy consultation room when speaking at a normal volume [31]. Providers should consider how to audit whether this guidance is followed. Further, to improve pharmacy users’ experience future research could explore how privacy could be better addressed in the pharmacy.

Some users in our study had negative experiences because staff were not respectful of users’ choices, not confident or were judgemental. A previous study showed that judgemental attitudes of healthcare professionals can negatively influence females’ experience when presenting for emergency contraception [32]. Further, respecting patient preferences and being confident are associated with a positive patient experience [33]. Pharmacy staff training should therefore focus on these specific areas.

The sex of the consulting pharmacist was important to pharmacy users. Some users reported a preference for same sex service delivery, whilst others preferred to be counselled by someone of the opposite sex. While some users did not indicate a reason for their preference, others stated that their preference was related to their sexuality. The preference for a specific sex of staff when examining intimate body parts in relation to pharmacy users’ sexuality was explored in a recent study [34]. Data from our study suggest that such preferences do not only exist where patients are physically examined but also where they discuss sexual and reproductive health. Potentially, creating more transparency on whether female or male pharmacists are working in pharmacies may increase the acceptability of sexual health services. This finding should be the focus of further research.

In line with our study, research from America and Australia found that language barriers were a concern for pharmacy staff and sometimes a barrier to pharmacy-based services [35,36]. Language barriers can adversely affect patients in their comprehension and adherence, quality of care, and patient and provider satisfaction [37] and therefore are important to address. There is evidence to suggest that pharmacies using telephone interpreting services can improve communication with patients who have limited English proficiency [35]. Pharmacies in Australia have access to Australia’s Translating and Interpreting services, a service which provides access to on-site interpreting services in over 150 languages [36]. Improving interpreting services within pharmacies in England may assist in overcoming communication barriers.

In agreement with other research on extended pharmacy services, the delivery of SRHS added workload and sometimes time pressure for pharmacy staff, particularly through the collection of pharmacy users’ data and the consultation itself [38,39]. While the service level (Tier 1 or Tier 2) that pharmacists were working at was not associated with the experience of workload, the existing busyness of the pharmacy and level of staffing levels were important. Similar to what was found in previous studies, many pharmacy staff members in this study noted that delivering extended services is difficult when staffing levels are low [39]. The direct entry of data collected by pharmacy healthcare assistants and the opportunity to pre-register clients in advance to the consultation with the pharmacist are potential solutions to take pressure from pharmacists. Since data in this and other studies suggest that workload is a concern for many pharmacy staff members, policymakers and health providers should consider, including an assessment of workload prior to introducing new pharmacy services. Further, it may be helpful for pharmacies if policymakers provide guidance on how many pharmacy staff members are necessary to run specific services.

Some pharmacy users were uncomfortable providing their personal information in the consultation and for some this was a barrier to the uptake of *Umbrella*’s services. In addition to shortening the consultation time, pre-registration may also provide pharmacy users with more comfort, as they can enter their personal information privately. Pharmacy staff and users had mixed views on whether electronic patient data should be shared with other health providers, but access to health records has been found to be important to make patient care decision [26], and future research should explore whether this would be feasible, and acceptable.

Our study indicated that there was a lack of awareness for pharmacy-based SRHS. This mirrors what has been found in previous studies on other extended pharmacy services, suggesting that this may represent a common issue for extended pharmacy services in general [28]. To use pharmacies’ full potential, awareness has to be raised on where and how services can be accessed, e.g., through more advertisement and more accurate and detailed online information.

Some pharmacy users who participated in the interviews stated that they had experienced difficulties with collecting the blood sample as part of the STI self-sampling kits using the lancets, and some reported that they did not manage to complete collection of the required blood sample. In contrast, a study from the Netherlands, in which no lancets but dried blood spot testing was used to screen for syphilis, HIV and hepatitis B found that the majority of participants found it easy to complete the finger-prick test to collect their sample without prior training [40]. This suggests that blood testing through STI self-sampling kits can be feasible, but that health providers offering pharmacy-based STI self-sampling kits should ensure that the testing kits are simple and feasible for people to conduct.

Strengths and Limitations

A major strength of this study was that we interviewed all three groups (pharmacists, pharmacy healthcare assistants and pharmacy users) which are involved in the delivery of SRHS. This allowed a comprehensive insight into the delivery of pharmacy-based SRHS. Another strength of the study was that data saturation was achieved and clear themes identified for both pharmacy staff and pharmacy users. The data collected were rich, and we were able to capture different experiences of pharmacy-based SRHS. However, given that interviews were exclusively conducted with pharmacy staff and pharmacy users from Birmingham (West Midlands), it has to be acknowledged that the findings are not generalizable. However, they may be transferrable to other areas. Our recently published retrospective study showed that *Umbrella*’s pharmacy-based SRHS are used by a large range of demographic groups [41]. However, the sample in this interview study was of comparatively limited demographics, and this is another limitation of this study. For example, no pharmacy users under 18 or over 35, and no White/White British or Black/Black British males could be recruited, despite our intention to sample these groups. Future research should try to focus to capture views from a diverse sample.

## 5. Conclusions

This was the first study to look at pharmacists’, pharmacy healthcare assistants’ and pharmacy users’ experiences of a large range of SRHS. The findings suggest that pharmacy-based SRHS were convenient to use for pharmacy users and that pharmacy staff were motivated to provide those services. However, several areas related to the nature of the pharmacy, to pharmacy staff interpersonal skills and the implementation of SRHS were identified where improvement could help unleash pharmacies’ full potential.

## Figures and Tables

**Table 1:** Overview of *Umbrella’s* pharmacy services.

Type of *Umbrella* Pharmacy Service	*Umbrella*’s Pharmacy Services	Eligibility by Age	Eligibility by Sex	Tier
**Contraception Services**	Emergency Contraceptive Pill	13–60	Female	Tier 1 and Tier 2
	Referral or Appointment for the copper coil at closest sexual health clinic	13–60	Female	Tier 1 and Tier 2
	Oral Contraception	13–60	Female	Tier 2
	Contraceptive Injection	13–60	Female	Tier 2
	Condoms	≥13	Female and Male	Tier 1 and Tier 2
**STI Testing Services**	Collection of pre-ordered STI self-sampling kits	≥16	Female and Male	Tier 1 and Tier 2
	STI self-sampling kits provided directly to pharmacy users	≥16	Female and Male	Tier 2
	STI self-sampling kit testing for chlamydia and gonorrhoea only	15–24	Female	Tier 2
**STI Treatment Service**	Chlamydia Treatment	≥13	Female and Male	Tier 2

**Table 2:** Pharmacy staff characteristics.

Pharmacy Staff Characteristics	Pharmacists		Pharmacy Healthcare Assistants	
	Gender		Gender	
	Female	Male	Female	Male
Total, *n*	4	5	5	1
Age				
<30	3	2	1	-
30–39	1	2	2	-
40–49	-	1	-	1
≥50	-	-	2	-
Ethnicity				
White/White British	-	1	3	1
Asian/Asian British	4	2	2	-
Black/Black British	-	2	-	-
Religion				
Christianity	-	-	2	-
Islam	2	3	1	-
Hinduism	-	1	-	-
Sikhism	2	-	-	-
No Religion	-	1	2	1
Type of *Umbrella* pharmacy employed at				
Tier 1	1	2	2	-
Tier 2	2	2	3	1
Tier 1 and Tier 2	1	1	-	-
Years in profession				
<5	3	2	1	-
5–9	1	-	1	1
10–19	-	2	2	-
20–30	-	1	1	-
Years in current role				
<5	4	2	1	1
5–9	-	2	2	-
10–19	-	1	1	-
20–30	-	-	1	-

**Table 3:** Pharmacy users’ characteristics.

Characteristics of Pharmacy Users	Gender	
	Female	Male
Total number, *n*	13 (one of those identified as transgender woman)	2
18–24	5	1
25–29	6	1
30–34	2	-
White/White British	3	-
Asian/Asian British	3	1
Black/Black British	3	-
Mixed/Multiple Ethnic Group	4	1
Christianity	3	-
Islam	2	-
Hinduism	-	1
Sikhism	-	-
No Religion	7	1
Prefer not to say	1	-
Emergency Contraception	8	-
Oral Contraception	2	-
Contraceptive Injection	1	-
Condoms	3	2
STI Self-Sampling Kits	4	
Chlamydia Treatment	3	1

## Appendix A

**Table A1:** Example questions from the topic guide.

Example Questions and Prompts Used in Semi-structured Interviews with Pharmacy Staff and Pharmacy Users
**Pharmacy User Interview**
**Can you tell me why you decided to go to the pharmacy to get (NAME OF THE SERVICE)?** **Prompts:** Have you obtained (NAME OF THE SERVICE) before? Have you used an Umbrella service before? Have you used an Umbrella service at a pharmacy before? How did you find out about Umbrella’s pharmacy services?
**Can you tell me about your experience of getting an Umbrella service at a pharmacy?** **Prompts:** What did you feel like when asking for the Umbrella service? How did you feel during the service delivery? How would you describe the pharmacy staff that offered or delivered the service?
**Pharmacy Staff Interview**
**Can you tell me about your experience delivering Umbrella’s services?** **Prompts:** What is it like asking pharmacy users for personal information? How do you feel when delivering sexual and reproductive health services? Are there any challenges when delivering sexual and reproductive health services?
**What impact does the delivery of Umbrella’s services have on you?** **Prompts:** How does the delivery of Umbrella’s services impact your routine work? How does the delivery of Umbrella’s services impact your career/ job satisfaction?

## References

[1] Rowley J., Hoorn S.V., Korenromp E., Low N., Unemo M., Abu-Raddad L.J., Chico R.M., Smolak A., Newman L., Gottlieb S. (2019). Chlamydia, gonorrhoea, trichomoniasis and syphilis: Global prevalence and incidence estimates, 2016. Bull. World Health Organ..

[2] Stulpin C., Marrazzo J.M. (2019). WHO: 1 million new urogenital STIs acquired each day worldwide. Infect. Dis. Child..

[3] Bearak J., Popinchalk A., Alkema L., Sedgh G. (2018). Global, regional, and subregional trends in unintended pregnancy and its outcomes from 1990 to 2014: Estimates from a Bayesian hierarchical model. Lancet Glob. Health.

[4] Aranda K., Coleman L., Sherriff N., Cocking C., Zeeman L., Cunningham E. (2017). Listening for commissioning: A participatory study exploring young people’s experiences, views and preferences of school-based sexual health and school nursing. J. Clin. Nurs..

[5] Public Health England (2018). Sexually Transmitted Infections and Screening for Chlamydia in England.

[6] Office of National Statistics (2018). Conceptions in England and Wales. https://www.ons.gov.uk/peoplepopulationandcommunity/birthsdeathsandmarriages/conceptionandfertilityrates/bulletins/conceptionstatistics/2014.

[7] Walker I., Leigh-Hunt N., Lee A. (2016). Redesign and commissioning of sexual health services in England—A qualitative study. Public Health.

[8] Ashby J., Ahmed N., Goldmeier D. (2019). Sexual difficulties service provision within sexual health services in the UK: A casualty of postcode lottery and commissioning?. Sex. Transm. Infect..

[9] Hind J. (2013). Commissioning Sexual Health Services and Interventions: Best Practice Guidance for Local Authorities.

[10] NHS Digital General Pharmaceutical Services in England 2008/09—Key Facts. https://digital.nhs.uk/data-and-information/publications/statistical/general-pharmaceutical-services/in-2008-09---2018-19-ns.

[11] Public Health England (2017). Sexual Health, Reproductive Health and HIV: A Review of Commissioning.

[12] Jewell S., Campbell K., Jaffer K. (2017). Umbrella: An innovative integrated sexual health service in Birmingham, UK. J. Fam. Plan. Reprod. Health Care.

[13] Gauly J., Ross J., Hall I., Soda I., Atherton H. (2019). Pharmacy-based sexual health services: A systematic review of experiences and attitudes of pharmacy users and pharmacy staff. Sex. Transm. Infect..

[14] Parker R.M., Bell A., Currie M.J., Deeks L.S., Cooper G., Martin S.J., Del Rosario R., Hocking J.S., Bowden F.J. (2015). ”Catching chlamydia”: Combining cash incentives and community pharmacy access for increased chlamydia screening, the view of young people. Aust. J. Prim. Health.

[15] Deeks L.S., Cooper G.M., Currie M.J., Martin S.J., Parker R.M., Del Rosario R., Hocking J.S., Bowden F.J. (2014). Can pharmacy assistants play a greater role in public health programs in community pharmacies? Lessons from a chlamydia screening study in Canberra, Australia. Res. Soc. Adm. Pharm..

[16] Downing S.G., Payze C., Doyle-Adams S., Gorton C. (2011). Emergency contraception over-the-counter: Practices and attitudes of pharmacists and pharmacy assistants in far North Queensland. Aust. N. Z. J. Obstet. Gynaecol..

[17] Ryder H., Aspden T., Sheridan J. (2015). The Hawke’s Bay Condom Card Scheme: A qualitative study of the views of service providers on increased, discreet access for youth to free condoms. Int. J. Pharm. Pract..

[18] O’Brien B.C., Harris I.B., Beckman T.J., Reed D.A., Cook D.A. (2014). Standards for reporting qualitative research: A synthesis of recommendations. Acad. Med..

[19] Patton M. Qualitative Research and Evaluation Methods. http://lst-iiep.iiep-unesco.org/cgi-bin/wwwi32.exe/[in=epidoc1.in]/?t2000=018602/(100).

[20] Hussainy S.Y., Stewart K., Chapman C.B., Taft A.J., Amir L.H., Hobbs M.K., Shelley J.M., Smith A.M. (2011). Provision of the emergency contraceptive pill without prescription: Attitudes and practices of pharmacists in Australia. Contraception.

[21] Marlow L.A.V., Waller J., Evans R.E.C., Wardle J. (2009). Predictors of interest in HPV vaccination: A study of British adolescents. Vaccine.

[22] Cooper R.J., Bissell P., Wingfield J. (2008). Ethical, religious and factual beliefs about the supply of emergency hormonal contraception by UK community pharmacists. J. Fam. Plan. Reprod. Health Care.

[23] Given L. (2008). The SAGE Encyclopedia of Qualitative Research Methods. http://sk.sagepub.com/reference/research.

[24] Green J., Thorogood N. (2018). Qualitative Methods for Health Research.

[25] Timonen V., Foley G., Conlon C. (2018). Challenges When Using Grounded Theory. Int. J. Qual. Methods.

[26] Famiyeh I.-M., MacKeigan L., Thompson A., Kuluski K., McCarthy L.M. (2019). Exploring pharmacy service users’ support for and willingness to use community pharmacist prescribing services. Res. Soc. Adm. Pharm..

[27] Watson M.C., Silver K., Watkins R. (2019). How does the public conceptualise the quality of care and its measurement in community pharmacies in the UK: A qualitative interview study. BMJ Open.

[28] Hindi A.M.K., Schafheutle E.I., Jacobs S. (2018). Patient and public perspectives of community pharmacies in the United Kingdom: A systematic review. Health Expect..

[29] Le P.-P., Braunack-Mayer A. (2019). Perspectives on privacy in the pharmacy: The views of opioid substitution treatment clients. Res. Soc. Adm. Pharm..

[30] Dhital R., Whittlesea C.M., Norman I.J., Milligan P. (2010). Community pharmacy service users’ views and perceptions of alcohol screening and brief intervention. Drug Alcohol Rev..

[31] NHS England (2019). Advanced Service Specification—NHS Community Pharmacist Consultation Service. https://www.england.nhs.uk/wp-content/uploads/2019/10/advanced-service-specification-nhs-pharmacist-consultation-service.pdf.

[32] Mooney-Somers J., Lau A., Bateson D.J., Richters J., Stewart M., Black K.I., Nothnagle M. (2018). Enhancing use of emergency contraceptive pills: A systematic review of women’s attitudes, beliefs, knowledge, and experiences in Australia. Health Care Women Int..

[33] Hindi A.M.K., Schafheutle E.I., Jacobs S. (2019). Applying a whole systems lens to the general practice crisis: Cross-sectional survey looking at usage of community pharmacy services in England by patients with long-term respiratory conditions. BMJ Open.

[34] Poria Y., Coyle A., Desombre T. (2019). Chapter Fourteen Serving all the Community? The Views and Preferences of Lesbian and Gay Consumers of Health Care. Qual. Health Care Strateg. Issues Health Care Manag..

[35] Bradshaw M., Tomany-Korman S., Flores G. (2007). Language barriers to prescriptions for patients with limited English proficiency: A survey of pharmacies. Pediatrics.

[36] Fejzic J., Barker M. (2019). Pharmacy practitioners’ lived experiences of culture in multicultural Australia: From perceptions to skilled practice. PLoS ONE.

[37] Schwei R.J., Del Pozo S., Agger-Gupta N., Alvarado-Little W., Bagchi A., Chen A.H., Diamond L., Gany F., Wong D., Jacobs E.A. (2016). Changes in research on language barriers in health care since 2003: A cross-sectional review study. Int. J. Nurs. Stud..

[38] Hilverding A.T., Mager N.A.D. (2017). Pharmacists’ attitudes regarding provision of sexual and reproductive health services. J. Am. Pharm. Assoc..

[39] Hindi A.M.K., Jacobs S., Schafheutle E.I. (2019). Solidarity or dissonance? A systematic review of pharmacist and GP views on community pharmacy services in the UK. Health Soc Care Community.

[40] Van Loo I.H.M., Dukers-Muijrers N.H.T.M., Heuts R., van der Sande M.A.B., Hoebe C.J.P.A. (2017). Screening for HIV, hepatitis B and syphilis on dried blood spots: A promising method to better reach hidden high-risk populations with self-collected sampling. PLoS ONE.

[41] Gauly J., Atherton H., Kimani P.K., Ross J. (2020). Utilisation of pharmacy-based sexual and reproductive health services: A quantitative retrospective study. Sex. Transm. Infect..

